# Epigallocatechin Gallate-Modified Gelatins with Different Compositions Alter the Quality of Regenerated Bones

**DOI:** 10.3390/ijms19103232

**Published:** 2018-10-19

**Authors:** Eiki Hara, Yoshitomo Honda, Osamu Suzuki, Tomonari Tanaka, Naoyuki Matsumoto

**Affiliations:** 1 Department of Orthodontics, Osaka Dental University, 8-1, Kuzuhahanazonocho, Hirakata, Osaka 573-1121, Japan; hara-e@cc.osaka-dent.ac.jp (E.H.); naoyuki@cc.osaka-dent.ac.jp (N.M.); 2 Institute of Dental Research, Osaka Dental University, 8-1, Kuzuhahanazonocho, Hirakata, Osaka 573-1121, Japan; 3 Division of Craniofacial Function Engineering, Tohoku University Graduate School of Dentistry; 4-1 Seiryomachi, Aobaku, Sendai 980-8575, Japan; suzuki-o@m.tohoku.ac.jp; 4 Graduate School of Science and Technology, Kyoto Institute of Technology, Matsugasaki, Sakyo-ku, Kyoto 606-8585, Japan

**Keywords:** bone quality, EGCG, bone formation, gelatin, collagen, FTIR imaging, picrosirius red staining, regenerated bone, polarized microscope

## Abstract

Bone quality is a significant indicator of the result of bone treatments. However, information regarding the quality of regenerated bones is limited. The study investigates the effect of different compositions of vacuum heated epigallocatechin gallate-modified gelatins sponge (vhEGCG-GS) on the quality of regenerated bones in critical size defects (9 mm) of rat calvariae. Five different compositions of vhEGCG-GSs containing the same amount of EGCG and different amounts of gelatin were tested. Following four weeks after implantation, the harvested regenerated bones were evaluated by using micro-computed tomography analysis, histological evaluation (hematoxylin-eosin and Villaneueva Goldner staining), picrosirius red-staining with polarized microscopic observation for collagen maturation, and Fourier transform infrared spectroscopy microscopy and imaging analysis for mineral-matrix ratio. The results indicated that increasing content of gelatin in the vhEGCG-GSs promoted bone and osteoid formation but yielded porous bones. Furthermore, tissue mineral density decreased and the maximum mineral-matrix ratio increased. In contrast, vhEGCG-GSs containing smaller amount of gelatin formed mature collagen matrix in the regenerated bones. These results suggest that the alteration of composition of vhEGCG-GSs affected the bone forming capability and quality of regenerated bone and provides valuable insight for the fabrication of new bone substitute materials.

## 1. Introduction

Bone is an organized tissue composed of rigid inorganic biological apatite and flexible bone matrix such as type 1 collagen [1]. Bone formation in large bone defects attributed to trauma, cancer, or congenital anomaly poses a challenge in dentistry and maxillofacial, plastic, and orthopedic surgery [2,3,4]. Currently, various biomaterials such as calcium phosphate, natural and artificial polymers, growth factors, and their combinations have been applied to assess their effectiveness in bone regeneration therapy [4,5,6,7]. Numerous studies have reported that most biomaterials can increase the bone mass through osteoconduction or osteoinduction when properly used. However, major determinants of bone fragility and strength include bone quality in addition to bone mass and bone mineral density (BMD) [8]. Bone quality has attracted wide attention clinically and has been used to diagnose osteoporosis, fracture risk, and the need for dental implants [9,10]. In 2000, NIH defined bone quality as “the sum of all characteristics of bone that influence the bone’s resistance to fracture,” which is distinct from BMD. Various parameters have been proposed to assess bone quality such as bone architecture (porosity), bone turnover, bone mineralization, and micro damage accumulation [10,11]. These parameters account for the mineralization, collagen status, mineral-matrix ratio, and chemical stability of hydroxyapatite [10,11]. Although multiple papers have reported on the bone quality of regenerated bones [5,10,12,13,14], a further detailed investigation will deepen our understanding of the regenerated bone. In particular, information on the collagen status in regenerated bone is still sparse. Moreover, the mechanism by which biomaterials affect the quality of regenerated bones remains unclear.

Epigallocatechin gallate (EGCG), which is a major component of the green tea polyphenol, is a promising health care agent [15]. This polyphenol is a safe and cost-effective agent and is known for its diverse pharmacological effects such as an antioxidant [16], anti-bacterial [17], anti-viral [18], anti-senescence [19], and anti-inflammatory effects [20]. Usage of EGCG in the medical field has been widely investigated [21]. In bone biology, this small molecule exhibits the ability to induce osteoblast differentiation in bone marrow mesenchymal stem cells [22] and dedifferentiated fat cells [23] and it elevates the activity of osteoblasts [24]. Nevertheless, information on the bone regeneration induced by EGCG in vivo has been limited. Recently, our group fabricated an EGCG modified gelatin sponge (EGCG-GS in which gelatin was chemically modified with EGCG) and reported its bone forming ability in the critical sized defect of mouse calvaria. EGCG-GS exhibited a superior bone forming ability than the gelatin sponge [25]. Additionally, we recently demonstrated that vacuum heated EGCG-GS (vhEGCG-GS) facilitated superior bone forming capability when compared with EGCG-GS and vacuum heated gelatin sponge in rat calvaria [26]. Therefore, vhEGCG-GS can be utilized as a usable model material to facilitate sufficient bone regeneration instead of the intact gelatin sponge and the vacuum heated gelatin sponge due to its superior bone forming ability. However, information regarding the quality of regenerated bone formed by vhEGCG-GSs is not available. Greater understanding of the bone quality of regenerated bones will provide valuable insight for advancement in bone regeneration therapy. 

The present study was designed to investigate the effect of different compositions of vhEGCG-GSs on bone forming capability and quality of regenerated bones. Multiple compositions of vhEGCG-GSs containing different amounts of gelatin and the same amount of EGCG were synthesized and implanted into critical sized defects of rat calvariae. Bone histomorphometric data of the treated calvaria including bone volume, two BMDs (volumetric bone mineral density: vBMD and tissue mineral density: TMD), and osteoid formation were evaluated by using micro-computed tomography μCT) analysis and/or histological evaluation (hematoxylin-eosin [H-E] and Villanueva Goldner [V.Goldner] staining). Collagen maturation and the maximum mineralization-matrix ratio were assessed by using picro-sirius-red staining with polarized microscopic observation and Fourier-transform infrared (FTIR) microscopy and imaging analysis, respectively. 

## 2. Results

### 2.1. Characterization of Prepared Sponges

Five types of vhEGCG-GSs (Table 1) were tested, which were designated as vhEGCG-GS[x] where x represented the amount of gelatin at synthesis. vhEGCG-GS[0.01] contained the smallest amount of gelatin. It did not exhibit spongy morphology and was, thereby, eliminated from further experimentation. 

**Figure 1:**
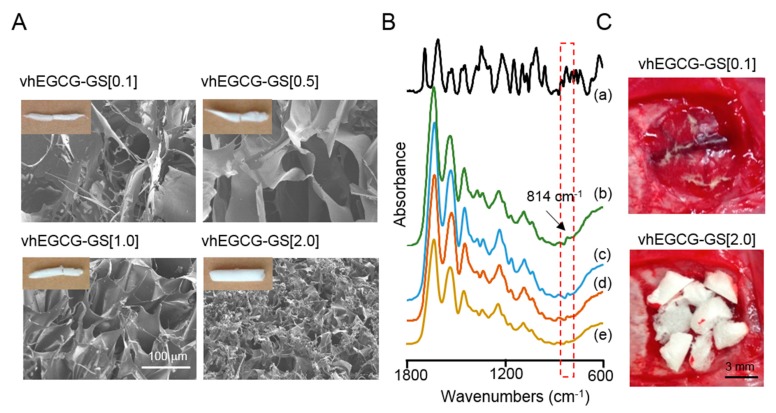
(**A**) Macroscopic and field emission scanning electron microscopic images of vacuum heated epigallocatechin gallate-modified gelatins sponges (vhEGCG-GSs). (**B**) Attenuated total reflection Fourier-transform infrared (FTIR) spectroscopic spectra of sponges. a: epigallocatechin gallate, EGCG, b: vhEGCG-GS[0.1], c: vhEGCG-GS[0.5], d: vhEGCG-GS[1.0], e: vhEGCG-GS[2.0]. (**C**) Representative implantation images of vhEGCG-GS[0.1 and 2.0] in defect immediately after implantation.

Macroscopic images of vhEGCG-GS[0.1 to 2.0] are illustrated in Figure 1A (upper left). All sponges exhibited similar length while the thickness of the sponges gradually increased in a dose-dependent manner with an increase in gelatin. The porous structure of the sponges was evaluated by using a field emission scanning electron microscope (FE-SEM). The pore size of vhEGCG-GSs gradually decreased with an increase in the content of gelatin (Figure 1A). The spectra of vhEGCG-GSs were analyzed by using attenuated total reflection FTIR spectroscopy to confirm the existence of EGCG. Specific EGCG peak at 814 cm^−1^ was identified in the spectra of all vhEGCG-GSs compositions [27]. These results suggest that all vhEGCG-GSs compositions successfully contained EGCG even though their architecture was different. 

### 2.2. Bone Formation in Critical Sized Defects of Rat Calvaria

**Figure 2:**
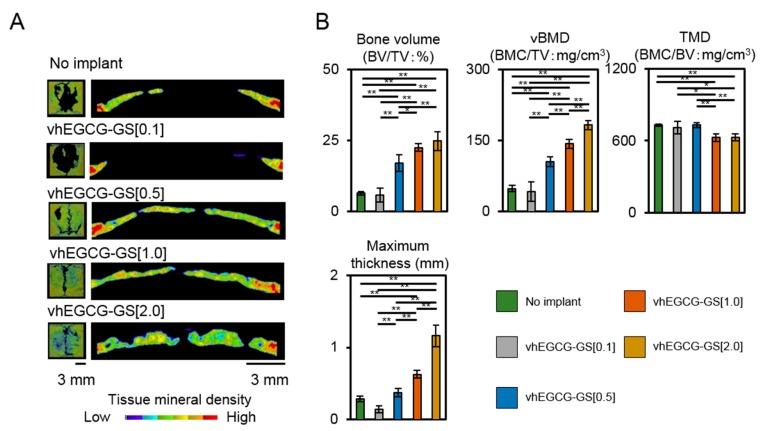
(**A**) Vertical and lateral tissue mineral density image of the defects with or without vhEGCG-GSs treatment after 4 weeks of implantation. No implant indicates bone defect without any implantation of sponges. (**B**) Histomorphometric data of defects: Bone volume (BV) per total volume (TV); vBMD: Bone mineral content (BMC) per TV, TMD: BMC/BV, Maximum thickness of mineralized tissue. * *p* < 0.05; ** *p* < 0.01 (analysis of variance (ANOVA) with Tukey-Kramer test). Data shows mean with standard deviation (S.D.) (*n* = 4, 4 rats per group).

**Figure 3:**
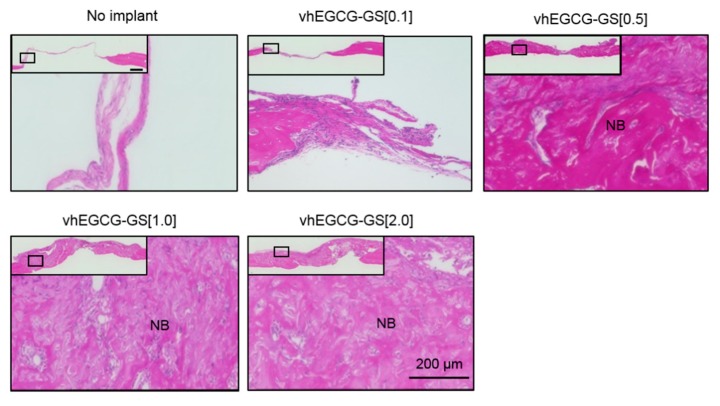
Hematoxylin-eosin (H-E) staining of defects with or without vhEGCG-GSs treatment after 4 weeks of implantation (low and high magnification images). Square in low magnification images indicates magnified area. NB: newly formed bone. Bar in low magnified area: 800 μm.

The critically sized defect (9 mm) of calvaria in Sprague Dawley rats (male, eight weeks old) was used to prepare the regenerated bone formed by vhEGCG-GSs. Following surgery, vhEGCG-GS[0.1] readily shrank due to the body fluid and was, thereby, unable to cover the defect while all other sponges covered the defects (Figure 1C). Figure 2 represents the tissue mineral density image and bone histomorphometric data of defects treated with or without sponges after 4 weeks of implantation. The implantation of vhEGCG-GS[0.1] and negative control did not form remarkable radiopacity in the defects. However, implantations with vhEGCG-GS[0.5, 1.0, and 2.0] mostly covered the entire defects within 4 weeks (Figure 2A). H-E staining verified that the radiopacity images were newly formed bone tissue (Figure 3). The bone volume (bone volume/total volume: BV/TV), vBMD (bone mineral contents/TV), and maximum thickness of the regenerated bone increased with incremental doses of gelatin in the vhEGCG-GSs. Meanwhile TMD (BMC/BV) slightly decreased in the defects treated with vhEGCG-GS[1.0 and 2.0]. These results suggest that the amount of gelatin in vhEGCG-GSs sensitively affected the bone formation and BMDs. 

### 2.3. Porosity of Regenerated Bone

**Figure 4:**
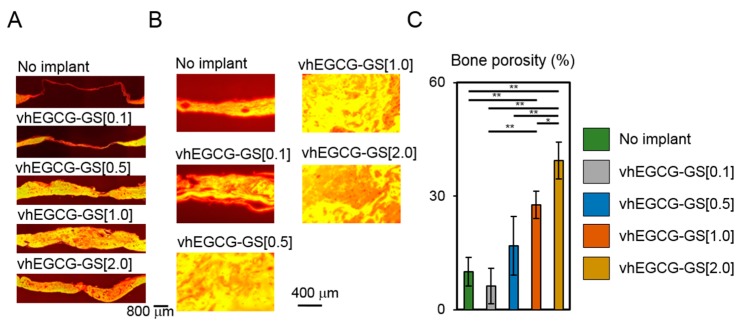
Fluorescent image (**A**,**B**) and quantitative data (**C**) of H-E stained defects with or without vhEGCG-GSs after four weeks of implantation. (**A**) Low magnification of treated calvaria. (**B**) High magnification of treated calvaria. Red: connective tissue or cavity (non-bone substrate area: bone marrow, gelatin, and connective tissue) in bone. Yellow: bone substrate area. (**C**) Bone porosity. Bone porosity was calculated as bone cavity (red) per total area (yellow plus red). * *p* < 0.05, ** *p* < 0.01 (ANOVA with Tukey-Kramer test). Data shows the mean with S.D. (*n* = 4: region of interest [ROI] from four rats per group).

A previous study reported that bone substrate and no-bone substrate can be distinguished on the basis of the fluorescence image of H-E staining [28]. We evaluated the porosity of the regenerated bones employing this technique. Figure 4A,B illustrates the fluorescence image of defects with and without sponge treatments. Cavities containing gelatin, bone marrow, and connective tissue were observed as red and bone substrate was observed as yellow in the images. An increased amount of gelatin in vhEGCG-GSs significantly elevated the cavity (red) in the regenerated bone, which suggests that the amount of gelatin in vhEGCG-GS altered porosity of the regenerated bone (Figure 4C).

### 2.4. Osteoid Formation

**Figure 5:**
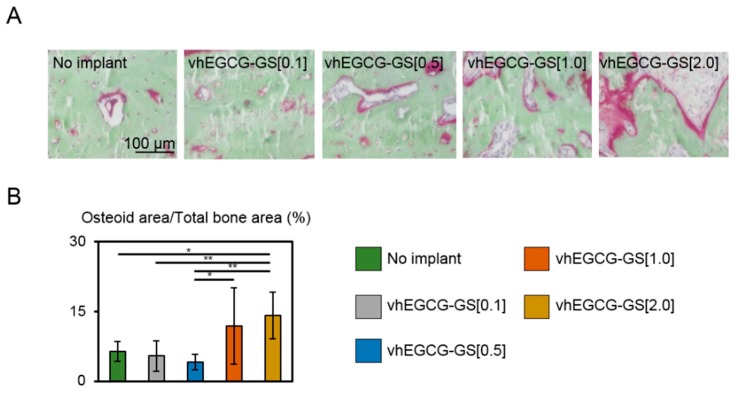
(**A**) Villanueva Goldner [V.Goldner] staining and (**B**) related quantitative data of defects with and without vhEGCG-GSs treatment after four weeks of implantation. (**A**) Red: osteoid. Green: mineralized bone tissue. (**B**) Osteoid volume: osteoid area (red) per total bone area (red plus green). * *p* < 0.05, ** *p* < 0.01 (ANOVA with Tukey-Kramer test). Data shows the mean with S.D. (*n* = 8: ROI from two rats per group).

V.Goldner staining was used to distinguish osteoid and mineralized bone tissue. Osteoid was observed as red and mineralized bone tissue was observed as green [29]. Furthermore, we evaluated the osteoid volume in regenerated bones after four weeks of implantation (Figure 5). Osteoid tissue was higher in defects treated with vhEGCG-GS[1.0 and 2.0] than defects treated with vhEGCG-GS[0.1 and 0.5] and defects without implantation, which suggests that the bone formation was more advanced in the defects treated with vhEGCG-GS[1.0 and 2.0].

### 2.5. FTIR Microscopic and Imaging Analysis

**Figure 6:**
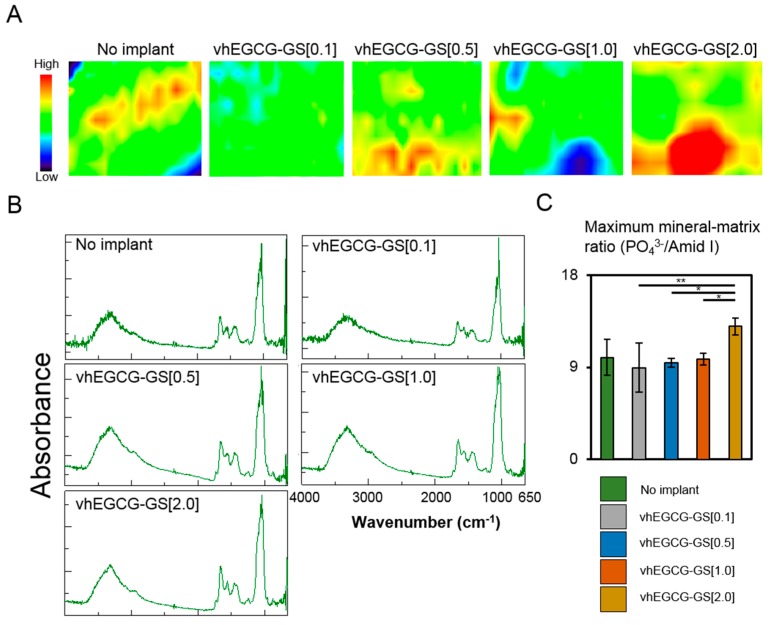
FTIR image (**A**), spectra (**B**), and quantitative data (**C**) of regenerated bone in the defects treated with or without vhEGCG-GSs after four weeks of implantation. (**A**) Distribution of mineral-matrix ratio: PO_4_^3−^/amide I. Color scale: PO_4_^3−^/amide I ratio calculated using pixel values. Scanning area: 187.5 μm × 100 μm. (**B**) Representative spectra of the point exhibiting maximum data of mineral-matrix ratio in the images. Mineral area (PO_4_^3−^; 915–1215 cm^−1^). Matrix area (Amide I; 1601–1714 cm^−1^). (**C**) Quantitative data of maximum mineral-matrix ratio. * *p* < 0.05; ** *p* < 0.01 (ANOVA with Tukey-Kramer test). Data shows the mean with S.D. (*n* = 4: ROI from two rats per group).

Based on the BMDs data from μCT analysis, we hypothesized that the maximum mineral-matrix ratio of the regenerated bone would change with the composition of the implanted sponge. Figure 6A shows FTIR images of the mineral-matrix ratio of the regenerated bones. Figure 6B depicts the spectra of maximum mineral-matrix ratio in the images. The maximum mineral-matrix slightly increased in the bone regenerated by vhEGCG-GS[2.0] when compared with other samples (Figure 6C).

### 2.6. Picrosirius Red Staining and Polarized Microscopic Observation

**Figure 7:**
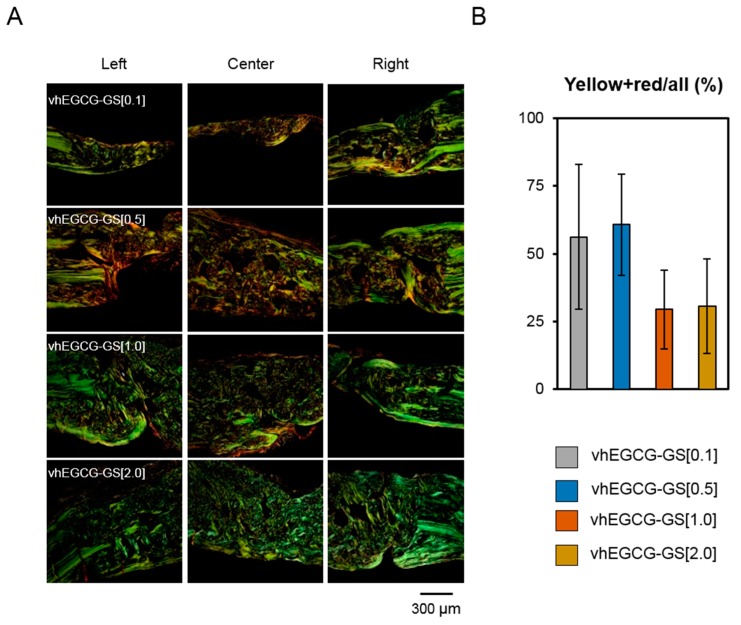
Picrosirius-red stained sections observed under polarized light (**A**) and quantitative data (**B**) of the defects treated with vhEGCG-GSs after four weeks of implantation. Green: immature (thin) type 1 collagen. Yellow-red: mature (thick and packing) type 1 collagen. (**B**) Ratio of mature collagen (yellow-red) per total collagen (yellow, green, and red). (ANOVA with Tukey-Kramer test). Data shows the mean with S.D. (*n* = 6: 2 central parts and four marginal parts of the defects from two rats per group).

Previous studies have reported that picrosirius red staining with polarized microscopic observation can be used to distinguish the maturation (thickness and packing) of type 1 collagen in the bone tissue [30]. Yellow and red stains represent mature type 1 collagen and green represents immature type 1 collage. The mother bone of calvaria exhibited a green color in the present study (Figure 7), which was observed in a previous study [31]. Notably, although there was no statistical significance, regenerated bone formed by the vhEGCG-GS[0.1 and 0.5] contained more mature collagen (red and yellow) and vhEGCG-GS[1.0 and 2.0] induced regenerated bone containing higher content of immature type 1 collagen (green). 

## 3. Discussion

Despite advances in bone regeneration therapy and bone biology, little is known about the bone quality of regenerated bones. In the present study, we demonstrated that the compositions of vhEGCG-GSs containing different amount of gelatin and the same amount of EGCG altered bone formation as well as quality of the regenerated bone. In particular, porosity, osteoid formation, and collagen maturation changed with incremental doses of gelatin in the vhEGCG-GSs. 

In the present study, we implanted four compositions of vhEGCG-GSs. As with our previous studies, vhEGCG-GS[1.0] induced sufficient bone formation within four weeks in large (9 mm) critical sized defect in rat calvaria [26] while newly prepared vhEGCG-GS[0.1] showed a poor bone forming ability (Figure 2). This difference can be explained by the covering area of the implanted sponges, which can be attributed to the amount of gelatin. Implantations of vhEGCG-GS[0.5, 1.0, and 2.0] covered the entire defect. However, some uncovered spaces were noted after the implantation of vhEGCG-GS[0.1] due to shrinking (Figure 1C). Thus, vhEGCG-GS[0.1] provided an indication of the pharmacological effect of EGCG but did not provide a sufficient scaffold for the cells in the defect. It is occasionally reported that combinatory use of inducers such as growth factors with scaffolds show greater bone forming ability than the single use of inducers or scaffolds [32,33]. In our previous study, we confirmed that osteoconduction occurred onto vhEGCG-GS[1.0] from the mother bone in the rat calvaria defect model [26]. These results reconfirm that the supply of sufficient scaffolding for bone forming cells is important to elicit bone regeneration by implanted biomaterials in vivo. 

When compared to the structure of four implanted sponges, we predicted that vhEGCG-GS[1.0 and 2.0] induced less bone formation than vhEGCG-GS[0.5] because vhEGCG-GS[1.0 and 2.0] were more condensed sponges than vhEGCG-GS[0.5] (Figure 1). It is widely reported that small pores cause less bone formation [34,35]. However, the regenerated bones created by vhEGCG-GS[1.0 and 2.0] recorded greater bone volume, vBMD, osteoid formation, and more porous structure than vhEGCG-GS[0.5] (Figure 2, Figure 4, and Figure 5). These discrepancies may be partially ascribed to the difference in stiffness of the sponges, which affects the shape. The sponges in the defects of the calvaria receive some sort of intracranial pressure from the brain [36]. Previous studies reported that an increased amount of gelatin enhanced the stiffness of the scaffold [37]. Consequently, increasing the amount of gelatin could have elevated the stiffness of sponges and retained the shape of sponges, which, thereby, yielded enough of a scaffold to facilitate bone forming for vhEGCG-GS[1.0 and 2.0]. As mentioned above, the bone formation by vhEGCG-GSs occurs when there is sufficient scaffolding for the cells. Furthermore, the maximum thickness of the regenerated bone increased with an increasing dose of gelatin in vhEGCG-GSs (Figure 2).

Conventional BMDs data obtained using μCT analysis is not always identical to the mineralization-matrix ratio obtained using FTIR microscopy analysis [38]. This is due to the equations used to calculate vBMD and TMD in the μCT analysis, which are computed as BMC/TV and BMC/BV, respectively. Additionally, the mineral-matrix ratio represents the weight of mineral per weight of matrix [8]. These differences disclose that the BMD accounts for the spatial spreading of minerals in the bone tissues or in total while the mineral-matrix ratio represents an intimate quantitative relation between weights of the mineral against the matrix. In the present study, TMD of vhEGCG-GS[1.0 and 2.0] were slightly smaller than vhEGCG-GS[0.1 and 0.5]. Conversely, a maximum mineral-matrix ratio of vhEGCG-GS[2.0] was slightly larger than that of the other sponges. These results might suggest that the mineral of the regenerated bone induced by vhEGCG-GS[2.0] dispersed more spaciously than vhEGCG[0.1 and 0.5]. However, the mineral was more aggregated in the matrix at some parts. 

Picrosirius red staining with polarized microscopic observation is widely recognized as a technique to distinguish the type of collagens such as collagen type 1 as yellow and red and collagen type 3 as green [39]. This method has been used to distinguish the maturation (thickness and packing) of collagens [30,40]. In our results, the mother bone was colored green while bones regenerated by vhEGCG-GS[0.1 and 0.5] were yellow and red. Although alteration of collagen types in bones regenerated by different materials would be an attractive hypothesis, type 1 collagen was the most abundant protein in the bone matrix protein [41]. Considering the composition of calvaria and its stained data reported previously, it is difficult to conclude that the green color of calvaria obtained in the present study contained sufficient type 3 collagen rather than type 1 collagen. The difference in color of the regenerated bones in the present study will reflect the maturation of type 1 collagen.

Unfortunately, we could not verify the mechanisms underlying the difference in the content of maturated collagen in bones regenerated by different sponges. Kaku et al. reported that excess mechanical loading can induce excess type 1 collagen maturation in the periodontal ligament (yellow and red) [40]. We previously reported that excess compressive stress to the octacalcium phosphate collagen sponge attenuated its bone formation [42]. In the present study, vhEGCG-GS[0.1 and 0.5] formed a poor amount of regenerated bone containing mature collagen (yellow and red) while vhEGCG-GS[1.0 and 2.0] formed a superior amount of regenerated bone containing collagen identical to the mother bone (green). Different resistances of the sponges to mechanical stress may regulate the status of type 1 collagen in regenerated bones. 

The condition of collagen (collagen cross-linking) is intimately associated with the bone strength, which affects the fate of bone diseases such as osteoporosis [43]. In the present study, we demonstrated that different compositions of vhEGCG-GSs altered the quality of regenerated bones in particular the bone structure, mineralization, and collagen status. However, we could not verify the mechanism by which these differences altered the function and fragility of the regenerated bones. Our previous studies have demonstrated that different EGCG concentrations in vhEGCG-GS altered the bone forming capability [26]. EGCG is known to modulate collagen production [44]. The results may indicate that the bone quality induced by vhEGCG-GS was affected by the concentration of gelatin as well as EGCG. Furthermore, we evaluated the regenerated bone during the early bone formation process. Dynamic bone remodeling occurred even after eight weeks of implantation in rat calvarial defects [45,46], which suggests that the quality of bone may change with time. Usable findings might be obtained by long term experiments tracking the bone quality of regenerated bones and comparative experiments between the native bone and regenerated bone after remodeling. Thus, further cautious experiments are essential to confirm the superiority of regenerated bones formed by vhEGCG-GSs. 

## 4. Materials and Methods

### 4.1. Materials

EGCG was purchased from Bio Verde Inc. (Kyoto, Japan), *N*-methylmorpholine (NMM) was purchased from Nacalai Tesque Inc. (Kyoto, Japan), 4-(4,6-dimethoxy-1,3,5-triazin-2-yl)-4-methyl-morpholinium chloride (DMT-MM) was purchased from Tokyo Chemical Industry Co., Ltd. (Tokyo, Japan), and type A gelatin from porcine skin was acquired from Sigma-Aldrich (St. Louis, MO, USA).

### 4.2. Synthesis of vhEGCG-GSs 

Five compositions of vhEGCG-GSs containing the same amount of EGCG but different amounts of gelatin were synthesized by applying aqueous chemical synthesis methods reported previously [26]. Type A gelatin (1, 10, 50, 100, or 200 mg) from porcine skin was dissolved in 5 mL warm Milli-Q water at 50 °C. Next, 0.07 mg EGCG, 27.5 μL NMM, and 69.2 mg DMT-MM were added to the gelatin solution and stirred in the dark for 24 h at room temperature. Dosages of gelatin and EGCG in the different compositions are listed in Table 1. The products were purified by dialysis using Spectra/Por7 MWCO 1000 (Spectrum Labs, Rancho Dominguez, CA, USA) in Milli-Q water in the dark. After dialysis for purification, the volume of the resultant aqueous solution including the product was adjusted to attain the desired gelatin concentration (0.01%, 0.1%, 0.5%, 1.0%, 2.0%) by the addition of Milli-Q water. The resultant solutions were poured into a silicon tubes (5 mm diameter, 7 cm height), which were stored for 24 h at -30 °C. Subsequently, the contents were lyophilized using DC800 (Yamato Co., Ltd., Tokyo, Japan) and then subjected to dehydrothermal treatment using vacuum heating with ETTAS AVO-250NS (AS ONE, Osaka, Japan) at 150 °C for 24 h with a gauge pressure of −0.1 MPa to obtain vhEGCG-GSs. All sponges were stored at 4 °C in the dark until use.

### 4.3. Characterization of Sponges

Macroscopic observation was performed by using Canon A495 camera (CANON, Inc., Tokyo, Japan). A FE-SEM (S-4800, Hitachi, Tokyo, Japan) was employed to confirm the porous structure of vhEGCG-GSs after the samples were coated with osmium using HPC-20 (Vacuum Device Ltd., Mito, Japan). SEM images were obtained at the condition of 5.0 kV and 10 μA. ATR-FTIR (IRAffinity-1S, Shimadzu, Kyoto, Japan) was utilized to confirm the presence of EGCG in vhEGCG-GSs over a range of 1800–600 cm^−1^ with 4 cm^−1^ resolution. The number of scans was 10. Data pre-processing algorithms were utilized to adjust the baseline and eliminate noise from the spectra by smoothing.

### 4.4. Implantation of Sponges

All animal experiments were approved by and strictly conformed to the guidelines of the Local Ethics Committee of Osaka Dental University (Approval No. 17-03005). Sprague Dawley rats (male, 8 weeks old) were anesthetized pre-operatively by using an intraperitoneal injection of a mixture of medetomidine hydrochloride, midazolam, and butorphanol tartrate. Critical-sized defect of the calvaria was prepared as reported previously [7]. An L-shaped incision was made in the parietal skin and periosteum to circumvent the effect of the surgery on the bone defect. Critical size defects (9 mm in diameter) were created in the center of the calvaria of each rat by using a trephine bar (Dentech, Tokyo, Japan). To attenuate the damage of bones, sterile saline was irrigated occasionally during the procedure. The defects were filled with vhEGCG-GSs (except for vhEGCG-GS[0.01]) and the periosteum and skin were overlaid and firmly sutured to stabilize the sponges. During the surgery, the cylindrical column of sponges was dissected indiscriminately (5 mm diameter, 2–3 mm height) and implanted in the defects. 

Control group comprised of rats without any implants. The rats were divided into the following groups: 1, No implant, 2, vhEGCG-GS[0.1], 3, vhEGCG-GS[0.5], 4, vhEGCG-GS[1.0], 5, vhEGCG-GS[2.0]. A total of 30 rats were used for the experiments (6 rats × 5 groups including a negative control for 4 weeks). At 4 weeks after the implantation, rats were sacrificed and the treated calvariae were harvested to examine the regenerated bones. 

### 4.5. Bone Histomorphometric Analysis using Microcomputed Tomography

After 4 weeks, the treated calvariae were harvested and fixed with a 10% formalin neural buffer solution (Wako, Tokyo, Japan) or ethanol. The samples were evaluated by using μCT (SMX-130CT, Shimadzu) at 47 kV and 47 μA radiation. Images were saved as 512 × 512 pixels. TRI/3D bone software (Ratoc Co, Ltd., Tokyo, Japan) was utilized to reconstruct the vertical and lateral views of the calvariae. To confirm mineralized tissue in the calvaria, TMD of the defect was visualized by using cylindrical phantoms containing hydroxyapatite (hydroxyapatite content: 200–1550 mg/cm^3^). BV/TV, vBMD (BMC/TV), and TMD (BMC/BV) of the defects were quantified to analyze the mineralized tissue volume, weight, and density. Lateral TMD images at the center of the defect were utilized to analyze the maximum thickness of the regenerated bone in the defects. Four rats per group were used for the bone histomorphometric analysis.

### 4.6. Hematoxylin–Eosin and Picrosirius Red Staining

Each fixed calvaria was decalcified using ethylenediaminetetraacetic acid solution, dehydrated, and embedded in paraffin. Thin sections (5 μm in thickness) were prepared and stained with H-E and a picrosirius red solution. H-E stained images were captured by using a BZ-9000 digital microscope (Keyence Co., Osaka, Japan). To assess the porosity of the regenerated bone, H-E stained samples were evaluated under a fluorescent condition using the TRITC filter. Bone substrate was observed as yellow while cavity (non-bone substrate: bone marrow, gelatin, and connective tissue) appeared as red images. Histomorphometric analysis [47] was applied to calculate the ratio of cavity (red) to the total volume (red plus yellow) in the regenerated bone using Adobe Photoshop Elements (Adobe Systems Inc., San Jose, CA, USA) and Image J (Image J 1.50i; NIH, Bethesda, MD, USA). The process was performed as follows: 1, capture the images of BZ-9000 digital microscope using TRITC filter, 2, trim the bone parts using Adobe Photoshop Elements (remove the non-bone part and periosteum), 3, prepare different figures of cavity in regenerated bone and of total bone (bone substrate and cavity in regenerated bone), 5, quantify the area by using Image J, and 6, calculate the ratio. A total of four regions of interest (ROIs) were quantified from four rats in each group. To distinguish the maturity of the collagen in the defect, picrosirius red stained images were captured by using polarized microscopy (Leica DFC300 FX, Leica Microsystems, Tokyo, Japan). As with the porosity assay, the ratio of mature (thick, yellow and red) per total collagen (yellow, red, and green) in the regenerated bone was calculated by using Adobe Photoshop Elements and image J. A total of six parts (two central parts and four marginal parts of the defects) were quantified from two rats for each group. 

### 4.7. V.Goldner Staining and FTIR Imaging 

Non-decalcified specimens after fixation were used for V.Goldner staining and FTIR microscopic and imaging analysis. The calvariae were sequentially dehydrated with graded ethanol series, acetone, and subsequently embedded in methylmethacrylate (MMA) resin. Thin sections (6 μm in thickness) were prepared by using microtome (RM2255, Leica Biosystems, Wetzlar, Germany) and stained with Villanueva Goldner stain for the V.Goldner staining. Stained sections were captured by using a BZ-9000 digital microscope. Mineralized bone was stained as green while non-mineralized osteoid tissue was stained as red. A total of eight ROIs were quantified from two rats in each group. For the FTIR microscopic and imaging analysis, after embedding in MMA, thin sections (3 μm in thickness) were prepared by using microtome and attached to the BaF_2_ slide. Each specimen was captured by using IRT-7000 (Jasco Engineering, Tokyo, Japan). Analysis conditions were: wave range, 4000–650 cm^−1^, resolution, 4 cm^−1^, and imaging pixel size, 187.5 × 100 μm. Mineral-matrix ratio (PO_4_^3−^/amide I) was calculated by integrating the phosphate band area (915–1215 cm^−1^) per amide I band area (1601–1714 cm^−1^) to compute the maximum mineral-matrix ratio. A total of four ROIs were selected randomly from two rats for each group to prepare FTIR images. The part representing a maximum mineral-matrix ratio from each image was used for quantitative analysis.

### 4.8. Statistical Analysis

The results are expressed as mean ± standard deviation (S.D.). One-way analysis of variance (ANOVA) was used to compare the means between the groups. If the ANOVA result was significant, the Tukey-Kramer test was used as a post hoc test. All statistical analyses were conducted by using an Excel package (Microsoft, Redmond, WA, USA) with add-in software Statcel 4 (OMS, Saitama, Japan). 

## 5. Conclusions

In the present study, we demonstrated that the alteration of composition of vhEGCG-GSs varied the quality of regenerated bones in critical-sized defect of rat calvariae. Increasing content of gelatin in vhEGCG-GSs elevated bone and osteoid formation yet yielded more porous bones. The alteration of gelatin amount affected the spatial distribution of minerals, the maximum mineral-matrix ratio, and collagen maturation in the regenerated bones. Although further cautious experiments are essential to conclude the superiority of regenerated bones in view of its function, the present findings clearly highlight that alteration of the composition of biomaterials affects the quality of regenerated bones. The findings will provide valuable insight during the preparation of biomaterials for new bone regeneration therapy.

## Figures and Tables

**Table 1:** Synthesis conditions to prepare the sponges.

Designation	EGCG (mg)	Gelatin (mg)	Water (mL)	Percentage of Gelatin (%)	Vacuum Heating	Used for Animal Experimentation
vhEGCG-GS[0.01]	0.07	1	10	0.01	+	No
vhEGCG-GS[0.1]	0.07	10	10	0.1	+	Yes
vhEGCG-GS[0.5]	0.07	50	10	0.5	+	Yes
vhEGCG-GS[1.0]	0.07	100	10	1	+	Yes
vhEGCG-GS[2.0]	0.07	200	10	2	+	Yes

## Abbreviations

GS	Gelatin sponge
vhGS	Vacuum heated gelatin sponge
EGCG	Epigallocatechin gallate
EGCG-GS	Epigallocatechin gallate-modified gelatin sponge
vhEGCG-GS	Vacuum heated EGCG-GS
